# Access to medications for opioid use disorder for formerly incarcerated individuals during community reentry: a mini narrative review

**DOI:** 10.3389/fpubh.2024.1377193

**Published:** 2024-05-13

**Authors:** Jason S. Chladek, Michelle A. Chui

**Affiliations:** ^1^Social and Administrative Sciences Division, School of Pharmacy, University of Wisconsin-Madison, Madison, WI, United States; ^2^Sonderegger Research Center for Improved Medication Outcomes, University of Wisconsin-Madison, Madison, WI, United States

**Keywords:** medication access, medications for opioid use disorder, opioid use disorder, formerly incarcerated, community reentry, transitions of care

## Abstract

Medications for opioid use disorder (MOUD) are especially important for formerly incarcerated individuals with opioid use disorder (OUD) and can reduce the risk of re-arrest and overdose during community reentry. Unfortunately, few formerly incarcerated individuals are able to access MOUD within the community, missing a critical tool for rehabilitation. A mini narrative review was conducted to highlight the published work that has been done to improve access to MOUD for formerly incarcerated individuals during reentry. The results yielded 15 records describing intervention evaluations, program descriptions, and research in progress. Most work is ongoing, showing promise that researchers have identified the importance of this problem. However additional research should be done to include other stakeholders and address the limitations of existing interventions and programs. Continued efforts can help ensure that formerly incarcerated individuals can safely and successfully reintegrate into society.

## 1 Introduction

Over three million U.S. citizens previously or currently suffer from opioid use disorder (OUD), a chronic disorder characterized by a problematic pattern of opioid use leading to health and social problems or distress (1, 2). Importantly, over 20% of people with OUD have been involved in the criminal justice system. Reports show that 20% of those in jail and 15% of those in prison are there for drug-based offenses, and an estimated two-thirds have a substance use disorder, with up to 25% having OUD (3). Medications for opioid use disorder (MOUD), including methadone, buprenorphine, and naltrexone, are a key component in the treatment of OUD. These medications can help block the euphoric effects of opioids and relieve physiological cravings (4). Due to the prevalence of OUD in correctional settings and importance of these medications in treatment, the availability of MOUD within jails and prisons has expanded over the last decade (5). However, continuation or initiation of MOUD within these systems is still limited. For example, in 2019, only 0.9% of confined inmates received some form of MOUD (6).

In addition, continuation or initiation MOUD is especially important for individuals during community transition. Formerly incarcerated individuals receiving MOUD are 85% less likely to die from drug overdose in the first month after release and have a 32% lower risk of re-arrest (7). However, few previously incarcerated individuals can access MOUD treatment upon reentry, missing a critical tool for rehabilitation and incurring a 40-fold greater likelihood of overdose following release compared to the general population (8). Previous work has shown that in individuals who are released with doses of MOUD, less than half continue use in the community, and individuals report significant barriers to continuity of care (8–11). Potential barriers include individual, community, and organizational factors, such as housing and transportation instability, stigma and discrimination, high cost, lack of insurance, or policies that treat MOUD as contraband. Previous research has also identified social barriers, including poor social support networks, and psychosocial barriers, including lack of motivation, competing priorities, and negative perceptions of MOUD (12–19). Clinical providers and correctional staff have identified high caseloads, limited understanding of MOUD, and lack of coordination between correctional and treatment systems as additional barriers (20–23).

Consequently, formerly incarcerated individuals account for up to 50% of overdose deaths in certain regions (24, 25). The Substance Abuse and Mental Health Services Administration (SAMHSA) also reports that 40–50% of these individuals are arrested for a new crime within a year of release, and 75% relapse to opioid use within 3 months (26). Re-arrest alone can negatively impact health outcomes by keeping those with OUD in correctional facilities, where conditions such as mental illness are often made worse (2). Additionally, involvement with the criminal justice system can block access to educational and employment opportunities, worsen mental health, decrease self-confidence, and lead to social withdrawal. Re-arrest can also negatively impact health and social outcomes for family members (27, 28). Furthermore, a lack of access to MOUD during community reentry is tied to racial and ethnic disparities, as Black, Hispanic, and Latine individuals are disproportionately impacted (29, 30).

The purpose of this review article is to summarize existing programs and interventions aimed at improving access to MOUD for formerly incarcerated individuals with OUD during community reentry. This review also aims to identify gaps within the programs and interventions and directions for future work.

## 2 Methods

A review was conducted to compile and synthesize published literature describing programs and interventions to improve access to MOUD for individuals transitioning from correctional settings to the community. The authors anticipated limited results and decided that a narrative mini review was the most appropriate method for summarizing this work and highlighting the limitations and gaps. Compared to other reviews, the objective of a narrative mini review is to summarize the most current and salient findings in a timely manner. The lead author (JC) identified keywords using a self-adapted version of the PICO model, as shown in Table 1 (31). These keywords were used to search relevant electronic databases, including PubMed, Scopus, and Web of Science. The search string is further detailed in the Supplementary material.

**Table 1:** Keywords used in literature search.

Population	Condition	Intervention	Environment
Prisoner Prison Jail Criminal Criminal justice system Corrections Correctional facility Incarceration Justice-impacted Justice-involved Formerly-incarcerated Previously-incarcerated	Opioid use disorder OUD Opioid addiction Opioid abuse	Medications for opioid use disorder Medications for OUD MOUD OUD treatment Medication-assisted treatment MAT Methadone Buprenorphine Naltrexone	Transition Community transition Reentry Re-entry Community reentry Community re-entry Decarceration Reintegration Post-incarceration Post-release

**Figure 1:**
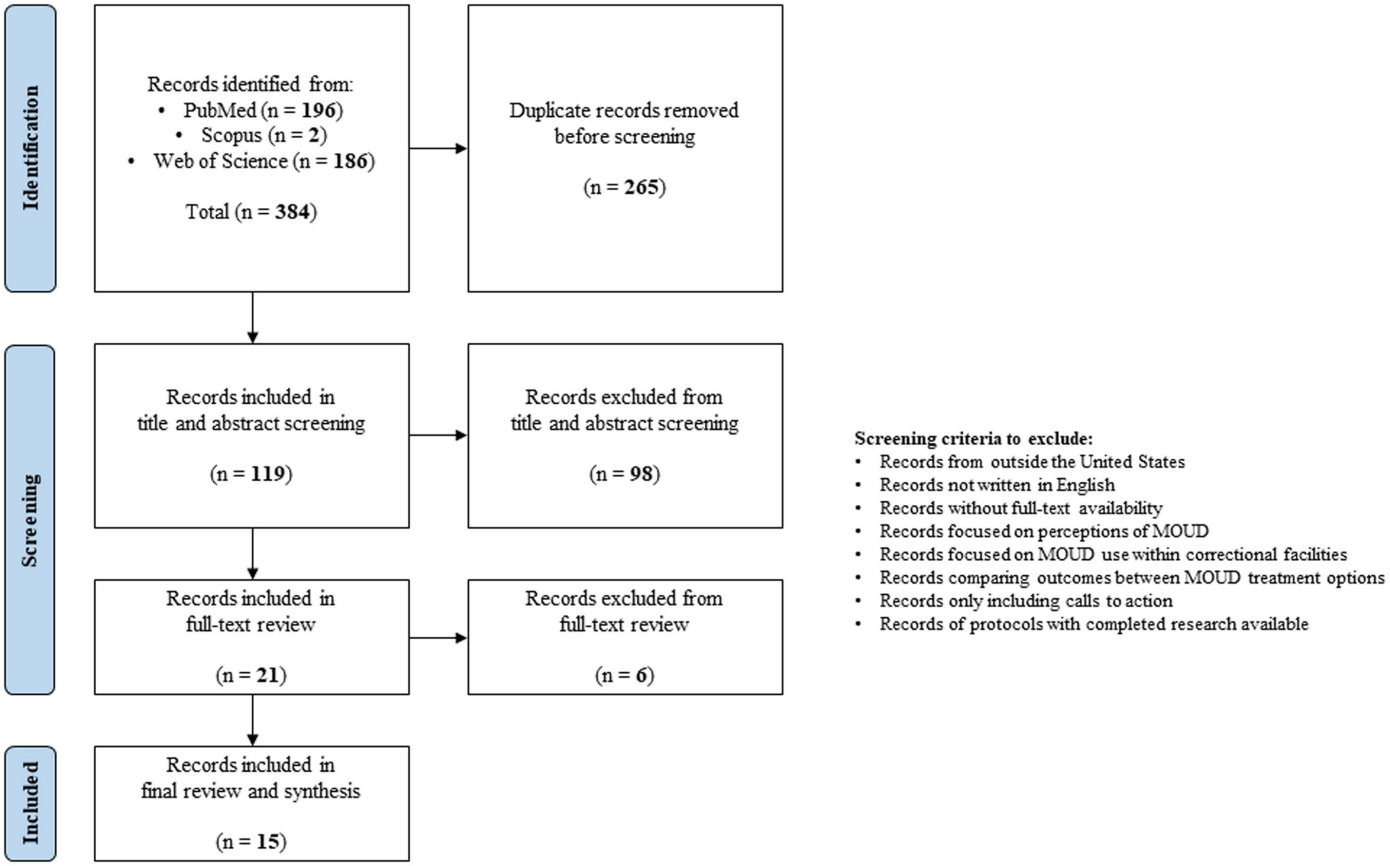
Literature search and selection strategy.

Study review and selection were also carried out by one reviewer (JC), as this was intended to be a rapid review. All records were compiled, and duplicates were removed. Other review articles were not included, but citations were searched for additional references. An initial screening of titles and/or abstracts was done to identify potentially relevant literature. Full-text articles were then selected based on the inclusion and exclusion criteria. Inclusion criteria included published peer-reviewed research articles and federally-published reports from the United States, written in English, indexed from inception to November 2023, and focused on programs or interventions to promote access to any form of MOUD for formerly incarcerated adults with OUD during community reentry. For this review, reentry could also include reentry through the parole system or other mechanisms for supervision. We also included records regardless of if participants were continuing or initiating MOUD within the community. Exclusion criteria included literature from outside the United States, written in languages other than English, or without full-text availability. Literature was also excluded if it only focused on evaluating perceptions of using MOUD, assessing MOUD continuation or initiation within correctional facilities, comparing outcomes between MOUD treatment options, or creating a “call to action.” Finally, study protocols were excluded if the completed research was available. The search and selection process is visualized in Figure 1.

Final literature was synthesized and presented in narrative format. Characteristics were identified, including literature type, setting and population, program or intervention components, and lead stakeholders involved.

## 3 Results

Table 2 summarizes the selected 15 records based on the characteristics outlined above. The records are categorized and described in more detail below.

**Table 2:** Summary of included records.

	Record type	Population/setting	Program or intervention components	Lead stakeholders involved
*Intervention evaluations*				
Farabee et al. (32)	Randomized controlled trial	135 jail inmates in the Bernalillo County Metropolitan Detention Center	Naltrexone injections with patient navigation	Physicians, patient navigators
Langdon et al. (33)	Feasibility and acceptability study	8 individuals from an outpatient primary care clinic in Rhode Island	Decisional balance exercises, distress tolerance coping skills, text messages	Counselors
Banta-Green et al. (34)	Feasibility study of pilot intervention	15 male participants from Washington State prisons and Department of Corrections community supervision	Education on OUD and available treatments, decision-making support, care navigation	Care navigators
Hanna et al. (35)	Formative qualitative implementation evaluation	Two state correctional facilities in Michigan	Case management, dual recovery therapy, peer support, vocational and educational supports, trauma-informed care, treatment planning	Case managers, peer support specialists
*Program descriptions*				
Krawczyk et al. (36)	Program description and initial outcomes	220 individuals at the Baltimore City Jail	Mobile low-threshold buprenorphine, transition to treatment program or primary care	Physicians, nurses, driver/site manager, peer recovery specialists
Tilson et al. (37)	Program description and initial outcomes	Women from six jail sites in Kentucky	Recovery assessments, recovery support sessions	Peer navigators
Substance Abuse and Mental Health Services Administration (26)	Program description	Individuals in the Rhode Island Department of Corrections	Medicaid enrollment assistance, treatment via Centers of Excellence, counseling, education, peer recovery support	Medical directors, project coordinators, program directors, clinicians, discharge planners, peer support specialists
Substance Abuse and Mental Health Services Administration (26)	Program description	Individuals in the Kentucky Department of Corrections	Naltrexone injections, cognitive behavioral therapy, relapse prevention support groups	Clinicians, case managers
Substance Abuse and Mental Health Services Administration (26)	Program description	Individuals in the Massachusetts state prisons	Personal recovery plans, oral and injectable naltrexone, care coordination and management	Clinicians, recovery support navigators
Substance Abuse and Mental Health Services Administration (26)	Program description	Individuals in custody at the Middlesex Sheriff’s Office (MSO) in Middlesex County, Massachusetts	Medicaid enrollment assistance, injectable naltrexone, care management	Clinicians, navigators
*Research in progress*				
Gordon et al. (38)	Study protocol	240 male and female prisoners from four prisons in Baltimore City and Baltimore County	Mobile naltrexone treatment	Nurses
Gordon et al. (39)	Study protocol	Male or female individuals on parole or probation in Baltimore	Buprenorphine via MedicaSafe dispensing devices	Physicians, nurses, addictions counselors
Howell et al. (40)	Study protocol	800 individuals from seven jails in Connecticut, New York, Puerto Rico, North Carolina, and Minneapolis	Enhanced primary care via Transitions Clinic Network	Clinicians, community health workers
Pho et al. (41)	Study protocol	1,000 individuals from rural and urban jails and prisons in Illinois	Connection to community services and OUD treatment, establishment of caseworker relationships, identification of goals, self-advocacy skill building, overdose education and naloxone distribution	Case managers, peer recovery coaches
Waddell et al. (42)	Study protocol	100 women from the Oregon Department of Corrections	Naltrexone injections, naloxone training and distribution, recovery mentorship	Certified recovery mentors, mental health counselors, nurses
Martin et al. (43)	Study protocol	680 individuals from seven community probation sites in Rhode Island, North Carolina, and Pennsylvania	Local change teams, peer support specialists	Parole officers, clinicians, case managers, administrative staff, peer support specialists
Scott et al. (44)	Study protocol	750 male and female individuals from 5 county jails in Illinois	Recovery management checkups	Linkage managers
Molfenter et al. (45)	Study protocol	48 jails and community-based treatment sites in Hawaii, Maine, Virginia, and Wisconsin	NIATx and ECHO coaching models	Criminal justice staff, health provider representatives, clinicians, counselors

### 3.1 Intervention evaluations

The results yielded one randomized controlled trial (RCT), which assessed the effectiveness of injectable naltrexone in conjunction with patient navigation. Patient navigation assisted participants in accessing care and overcoming barriers following release. This treatment was compared to naltrexone alone and enhanced treatment-as-usual with drug education. Primary outcomes included opioid use and meeting the criteria for OUD 6 months post-release, and the researchers found no significant differences by study condition for these outcomes (32).

Two articles reported intervention acceptability and feasibility. The first included an intervention utilizing clinician-delivered in-person meetings and text messages for 3 months after discharge. These components were designed to engage patients in decisional balance exercises, provide strategies to manage stress, drug cues, and psychological discomfort, promote ongoing MOUD engagement, and emphasize adaptive strategies for distress tolerance. Interviews indicated positive reactions toward the intervention, and participants believed the intervention to be generally viable (33). The second examined the feasibility of a pilot intervention to link participants to ongoing MOUD and psychosocial support. The intervention consisted of education on available OUD treatment, support with decision-making, and care navigation for 6 months post-release. Care navigation logs documented intervention engagement and service utilization, and follow-up interviews were conducted to assess satisfaction. Overall, the intervention had broad acceptability among participants and was feasible to implement. However, it did not demonstrate its intended effect to facilitate MOUD immediately post-release among the small sample size (34).

Finally, Hanna et al. conducted a formative qualitative evaluation to assess the fit of applying the Consolidated Framework for Implementation Research (CFIR) to a corrections and community-based opioid use treatment initiative. The initiative utilizes the six components of the evidence-based model, MISSION-CJ (Maintaining Independence and Sobriety through Systems Integration, Outreach, and Networking – Criminal Justice). The evaluation found CFIR to be a useful framework for understanding barriers and facilitators to implementation uptake of cross-system re-entry initiatives for individuals with OUD. Researchers found CFIR to be particularly valuable in reinforcing the use of implementation research as a way of continuous process improvement (35).

### 3.2 Program descriptions

Three records included descriptions of existing programs that help link individuals to MOUD treatment. The first described the development of Project Connection at Re-Entry (PCARE), which provides low-threshold buprenorphine treatment through a mobile van located outside the Baltimore City Jail. Treatment is provided by a primary care physician who prescribes buprenorphine, a nurse, and a peer recovery coach. Initial outcomes showed that in participants beginning treatment, 67.9% returned for a second visit or more, 31.6% were still involved in treatment after 30 days, and 20.5% were transferred to continued treatment at a partnering site (36).

The second described a program funded through the Justice Community Opioid Innovation Network (JCOIN), an initiative to connect investigators with justice and behavioral-health partners to improve care for individuals with OUD in justice settings. The program connects women in jail to peer navigators via videoconference. Navigators provide an initial reentry recovery assessment and 12 or more weeks of support sessions after reentry. Initial recovery assessments focus on discussions of needs/barriers, resources/supports, and recovery goals. The researchers also reviewed notes from initial sessions and conducted in-depth interviews with peers to document their perspectives on participants’ community transition. They discussed challenges and successes from the first year of the intervention. Notes showed that women anticipated challenges to reentry, and more than half chose OUD treatment as their primary goal. Specifically, 17.5% of participants mentioned a preference for MOUD post-release. In initial interviews, peers described transitions as unpredictable and discussed barriers related to stigma and establishing relationships via telehealth. However, they also discussed that peer navigation can offer critical linkages to services for women during release from jail (37).

Lastly, a resource guide from SAMHSA described four different re-entry programs. In Rhode Island, 12 MOUD “Centers of Excellence” were established. These centers were repurposed from an existing network of CODAC Behavioral Healthcare outpatient facilities. These facilities were scattered throughout the state and enabled formerly incarcerated individuals to continue MOUD regardless of their location post-release. In 2017, Rhode Island saw a 60.5% decrease in the overdose death rate among those recently incarcerated. In Kentucky, the Department of Corrections (DOC) helps fund Recovery Kentucky, which includes 14 addiction treatment sites across the state. These sites provide housing and continued treatment, including MOUD, post-release. Overall, 57.2% of individuals completing Recovery Kentucky had not been reincarcerated. Additionally, Massachusetts houses two major programs. Spectrum Health Services provides injectable naltrexone for individuals pre-release. On release, participants are directly referred to one of over 25 clinics maintained by Spectrum or provided through DOC partners. The program contributed to a 9.7% reduction in crime. In Middlesex specifically, the Sheriff’s Office implemented the Medication Assisted Treatment and Direct Opioid Recovery (MATADOR) program, which provides post-release treatment navigation and support. Navigators help guide individuals through treatment and communicate with participating community MOUD providers. To date, only 4.57% of participants had a fatal overdose after participation (26).

### 3.3 Research in progress

Results yielded a study protocol that will assess the impact of long-acting naltrexone injections post-release via mobile medical treatment at the patient’s place of residence Participants will be randomized to receive: (1) one injection of long-acting naltrexone in prison, followed by six monthly injections post-release at a community treatment program; or (2) one injection of long-acting naltrexone in prison, followed by six monthly injections post-release via mobile treatment. Primary outcomes will include treatment adherence, opioid use, criminal activity, re-arrest, re-incarceration, and HIV risk-behaviors (38). Another record describes the planned assessment of Buprenorphine Bridge Treatment (BBT) compared to treatment as usual. Under BBT, participants will begin buprenorphine using a MedicaSafe dispensing device, a tamper-resistant medication dispenser with an online platform that logs dispenses and adherence data. Treatment will continue until transition to a community program. Illicit opioid use and treatment adherence will be the primary outcomes of interest (39). A third team will assess whether follow-up care in a Transitions Clinic Network (TCN) will improve post-release opioid treatment outcomes. The TCN provides enhanced care by including a community health worker with a history of incarceration on the primary care team. The community health worker will focus on attending to the social needs of patients, including housing and food security. Researchers will randomize 800 individuals to a TCN or standard primary care and assess engagement in OUD treatment within 30 days of release (40).

Four of the protocols will evaluate professional or peer support. First, the Reducing Opioid Mortality in Illinois (ROMI) protocol describes a study to compare case management, peer recovery coaching, and overdose education and naloxone distribution (CM/PRC + OEND) to OEND alone. The intervention will involve linkage to treatment and support for continuity of care, skills building, and navigation of social service. The primary outcome will be engagement in MOUD (41). A second protocol describes the Reducing Overdose After Release from Incarceration (ROAR) pilot intervention. Participants will receive nasal naloxone, training on naloxone use, and regular check-ins with certified recovery mentors to facilitate sustained engagement with treatment. Mentorship will begin in the month prior to release and continue for 6 months in the community. Researchers will evaluate opioid overdose as the primary outcome (42). Third, another study will determine whether a facilitated local change team (LCT) intervention improves linkage to MOUD, and whether participant-level outcomes are enhanced by using peer support specialists (PSS). Participants will be randomized to receive PSS vs. treatment as usual. Those in the experimental arm will meet with a PSS for 12 months, and PSSs can help establish linkages to treatment, provide education, share skills, and set goals. The outcome of interest includes engagement in MOUD (43). Lastly, a research team will test an adapted version of the evidence-based Recovery Management Checkups (RMC), which provides MOUD linkage, support for retention, and re-linkage at quarterly checkups with a Linkage Manager. Individuals will either receive only Monitoring and Treatment Referral (MTR), quarterly RMC, or RMC-A, which adjusts the number and intensity of checkups based on an individual needs assessment at each checkup. Researchers will evaluate MOUD treatment initiation, engagement, retention, and relinkage (44).

Finally, a protocol outlines a trial for assessing two implementation strategies. This team will conduct a randomized controlled trial with 48 jails and community-based treatment provider sites that work with formerly incarcerated individuals with OUD. The trial will determine the optimal combination and dosages of two different coaching strategies: (1) The Network for the Improvement of Addiction Treatment (NIATx) model for process improvement, which provides technical assistance on MOUD implementation and organizational change to help organizations provide MOUD for justice-impacted patients; and (2) The Extension for Community Healthcare Outcomes (ECHO) model, which focuses on connecting clinical providers with expert MOUD prescribers to promote high-quality practices. The researchers will conduct exploratory analyses of baseline MOUD practices and changes over time (45).

## 4 Discussion

Overall, there is a clear need to improve MOUD access for this patient population. Yet, this review demonstrates that research to address this problem remains limited, and most work is in progress. Additionally, most intervention evaluations focus on acceptability/feasibility, rather than effectiveness, or are limited to certain geographic areas and sub-populations, such as male or female-only inmates. This review may also be limited by the inclusion and exclusion criteria and databases used in the search and selection process. For example, we did not include records that focused on strategies during incarceration, which may have offered additional insight for post-release strategies. There was also not enough information to evaluate records based on whether the participants were continuing or initiating MOUD upon reentry, which may be a factor influencing long-term MOUD use.

While the results are limited, and it is not possible to identify a one-size-fits-all solution, there are components of the existing interventions and programs that show promise. First, the use of mobile treatment showed positive outcomes associated with treatment adherence and maintenance (36). Second, text messages designed to engage patients in decisional balance exercises, stress management, and MOUD engagement were positively received and deemed viable (33). Third, programs demonstrated that education on MOUD helps improve long-term health and reincarceration outcomes when coupled with other treatment assistance (26). Finally, while the records showed mixed results, care and/or peer navigation show potential in facilitating treatment engagement within the community (26, 34, 37). Future efforts should scale up these interventions and programs and explore generalizability and long-term effectiveness.

Research should also explore how to address the limitations and challenges faced in these studies. For example, one program showed the positive impacts using peer navigators. However, patients still experienced barriers related to stigma and developing relationships via telehealth (37). Future work should explore how to reduce stigma related to MOUD and this patient population. Additionally, next steps should examine how to improve relationships between formerly incarcerated individuals and telehealth providers or navigators. Similarly, despite the promise of peer navigation as a resource, the RCT in this review showed no difference between naltrexone in conjunction with peer navigation compared to naltrexone alone. The researchers noted that this was likely due to the participants’ low rates of engagement with peer navigators and, potentially, other priorities (32). Future research should focus on how to help patients better engage with peer navigators and balance priorities. Notably, a focus on telehealth, patient relationships, and social support aligns with strategies deemed important by justice-impacted patients (46).

In looking at stakeholder involvement, another potential resource that has not been explored is community pharmacists. The success of mobile treatment demonstrates that location of MOUD can facilitate access (36). Community pharmacists are not only more accessible than other healthcare providers, but 96.5% of the U.S. population lives within 10 miles of a community pharmacy (47). Previous work has demonstrated that community pharmacists can face barriers to providing MOUD services, including problems related to fixed costs, reimbursement, time, and lack of training (48). With that in mind, future work could focus on addressing these barriers, as well as barriers that formerly incarcerated individuals face in utilizing community pharmacies.

As noted, most records in this review include research in progress. While this may limit our ability to draw conclusions, it shows that researchers are recognizing this problem. Continuing these efforts and addressing the gaps and limitations noted above can help formerly incarcerated individuals access MOUD and improve several public health outcomes. Importantly, improving community transition for individuals with OUD can help ensure that this population is not tossed aside and given the opportunity to successfully reintegrate into society.

## Author contributions

JC: Conceptualization, Data curation, Methodology, Writing – original draft, Writing – review & editing. MC: Conceptualization, Supervision, Writing – review & editing.
