# Supplementary material for: Access to medications for opioid use disorder for formerly incarcerated individuals during community reentry: a mini narrative review
Source: Front Public Health. 2024 May 13;12:1377193. doi: 10.3389/fpubh.2024.1377193 (PMC11128549; doi:10.3389/fpubh.2024.1377193)
Supplement: Supplementary file 1 [file Table_1.DOCX]

Supplementary Material

# Search string for all databases

(prisoner OR prison OR jail OR criminal OR criminal justice system OR corrections OR correctional facility OR incarceration OR justice-impacted OR justice-involved OR formerly-incarcerated OR previously-incarcerated) AND (opioid use disorder OR OUD OR opioid addiction OR opioid abuse) AND (medications for opioid use disorder OR medications for OUD OR MOUD or OUD treatment or medication-assisted treatment OR MAT OR methadone OR buprenorphine OR naltrexone) AND (transition OR community transition OR rentry OR re-entry OR community reentry OR community re-entry OR decarceration OR reintegration OR post-incarceration OR post-release)
